# Editorial: Seizures in brain tumors

**DOI:** 10.3389/fsurg.2024.1504572

**Published:** 2024-10-29

**Authors:** Mohammad Mofatteh, Saman Arfaie, Mohammad Sadegh Mashayekhi, Phillip L. Pearl, Sunit Das, Aaron Cohen-Gadol

**Affiliations:** ^1^ School of Medicine, Dentistry and Biomedical Sciences, Queen’s University Belfast, Belfast, United Kingdom; ^2^ Department of Neurosurgery, Neuro International Collaboration, London, United Kingdom; ^3^ Division of Neurosurgery, Department of Clinical Neurological Sciences, Schulich School of Medicine and Dentistry, University of Western Ontario, London, ON, Canada; ^4^ Department of Neurosurgery and Neurology, McGill University Faculty of Medicine, Montreal, QC, Canada; ^5^ Department of Neurosurgery, Neuro International Collaboration, London, ON, Canada; ^6^ Division of Neurosurgery, Faculty of Medicine, University of Ottawa, Ottawa, ON, Canada; ^7^ Department of Neurosurgery, Neuro International Collaboration, Ottawa, ON, Canada; ^8^ Department of Neurology, Boston Children’s Hospital and Harvard Medical School, Boston, MA, United States; ^9^ Division of Neurosurgery, Department of Surgery, St. Michael’s Hospital, University of Toronto, Toronto, ON, Canada; ^10^ Department of Neurosurgery, The Neurosurgical Atlas and Atlas Meditech, Carmel, IN, United States; ^11^ Department of Neurological Surgery, Keck School of Medicine of the University of Southern California, Los Angeles, CA, United States; ^12^ Department of Neurosurgery, Neuro International Collaboration, Los Angeles, CA, United States

**Keywords:** seizure, epilepsy, brain tumor, glioma, glioblastoma, anti-seizure medication, oligodendrogliomas, astrocytoma

**Editorial on the Research Topic**
Seizures in brain tumors

Epileptic seizures can be the first presenting symptom before a diagnosis is confirmed in approximately 30%–60% of primary brain tumor patients (1). In addition, seizures due to tumors of the central nervous system are the second most common cause of epilepsy in adults (following hippocampal sclerosis) or children (following focal cortical dysplasia) (2). Seizure-related complications can significantly affect clinical prognosis and directly affect quality of life, including mental health (3, 4). In comparison to metastatic lesions and high-grade tumors, such as glioblastoma, a higher seizure incidence is observed in low-grade tumors, especially in association with dysembryoplastic neuroepithelial tumors (DNET) and gangliogliomas, known as “long-term epilepsy-associated brain tumors” (LEATs), as well as oligodendrogliomas and low-grade astrocytomas (5, 6).

Various explanations, including long-term impairment of the subcortical network and denervation hypersensitivity, have been proposed as potential pathophysiologic mechanisms for seizure onset (7, 8). Conventional therapies, including surgery, radiotherapy, and chemotherapy can result in symptomatic control; however, brain-tumor-related epilepsies remain refractory to antiseizure medications (7, 8). Multiple factors, such as tumor subtype, WHO grade, and tumor location can influence the onset, frequency, and treatment resistance of seizures (9). Therefore, having a better understanding of the molecular mechanisms underlying brain-tumor-related seizures can provide insight into the treatment of patients. In this collection, we aimed to collate the most recent clinical and translational advances in the field to demonstrate advances in seizures associated with intracranial tumors.

A common complication of brain tumor surgery is early postoperative seizures (EPS), which can result in hemorrhage, elevated intracranial pressure, cerebral hypoxia, increased hospitalization, reduced quality of life, reduced survival, and higher morbidity (10). Investigating the occurrence and relationship between postoperative seizures and *de novo* epilepsy following craniotomy surgery was the main focus of a retrospective single-center cohort study by Horiuchi et al. Investigating 293 patients undergoing craniotomy, they found that the rate of EPS (defined seizure onset within 7 days post-surgery) was 3.8%, whereas the delayed postoperative seizure (DPS) (later than 7 days and less than 60 days post-surgery) rate was 3.3%. The rate of *de novo* postoperative epilepsy was 4.9%, and occurrence of EPS and DPS were significantly associated with *de novo* epilepsy. In this study, prophylactic administration of anti-seizure medication was not effective in preventing EPS or postoperative *de novo* epilepsy.

Despite complications associated with EPS, there are no formal guidelines on the management of perioperative seizures in patients with tumor-related epilepsy at risk of EPS (11). Freund et al. reported a case of a 38-year-old male diagnosed with isocitrate dehydrogenase-mutant mixed glioma who had a history of EPS that manifest with status epilepticus during prior tumor surgery. The patient underwent awake craniotomy, which is a method of choice for tumors in eloquent functional areas of the brain (12). Aggressive perioperative prophylactic anti-seizure medication therapy was adopted by using a higher maintenance dose of lacosamide and levetiracetam by 25% administered 48 hours prior to the surgery. In addition, intravenous infusion of fosphenytoin (20 mg/kg) prior to direct electrical stimulation, followed by a maintenance dosing of 300 mg/day for 14 days, prevented EPS occurrence. This case highlighted that aggressive prophylactic usage of anti-seizure medication therapy perioperatively could be considered in the management of patients with tumor-related epilepsy who are at risk of EPS. Future double-blinded randomized controlled trials are required to further validate and extend these observations. Also, the side effect profile of aggressive prophylactic anti-seizure medications and their effect on postoperative recovery need to be explored.

Seizure is a common presentation in patients with ganglioglioma; however, some patients still experience postoperative seizures. To gain a better understanding of seizure outcomes, Hu et al. conducted a retrospective analysis of 222 patients with a mean age at surgery of 19.19 ± 10.93 years who were diagnosed with ganglioglioma. The mean follow-up duration of 6.28 ± 3.17 years revealed that 78.4% of patients achieved class 1 or 2 seizure outcome, as evaluated by the International League Against Epilepsy (13). Conducting univariate and multivariate analyses revealed that seizure-free outcomes were associated with short duration of seizures and gross total resection, whereas bilateral interictal or ictal epileptiform discharges on preoperative video-electroencephalography were associated with poor outcomes, emphasizing the role of surgical resection as an effective method for epilepsy treatment associated with ganglioglioma.

Seizure presentation in patients with pituitary pathology is not a common phenomenon, and can occur due to electrolyte abnormalities (14). Instead, patients with pituitary tumors usually present with visual symptoms and headache due to mass effect on the optic chiasm and/or endocrine dysfunction (15). In their case report, Hong et al. describe a 54-year-old male with pituitary macroadenoma compressing the left temporal lobe who presented with a generalized seizure. His seizures resolved following endoscopic endonasal debulking of the tumor. This is a unique case that highlights the absence of visual symptoms or endocrine dysfunction in pituitary macroadenomas, where isolated seizures may be the sole presenting symptom. Seizure control can be achieved by relieving the mass effect on the temporal lobe through prompt surgical debulking.

In conclusion, seizures associated with central nervous system tumors remain an active area of research and articles included in this Collection can help push the boundaries of research and clinical practice. The collection consisted of two original research articles, which comprised 515 patients in total, and two case reports, demonstrating the diversity of research in this field. The insight presented in these articles holds promise for further investigations and improve patient outcomes. As we continue shedding light on the complications of brain tumors, we look forward to further investigation to improve patient outcomes and advance the practice of neurosurgery.
